# Attitude towards working in rural areas: a cross-sectional survey of rural-oriented tuition-waived medical students in Shaanxi, China

**DOI:** 10.1186/s12909-018-1209-z

**Published:** 2018-05-02

**Authors:** Jinlin Liu, Kun Zhang, Ying Mao

**Affiliations:** 1 School of Public Policy and Administration, Xi’an Jiaotong University, Xi’an, China; 2 Xi’an Health and Family Planning Commission, Xi’an, China

**Keywords:** RTME, Medical students, Attitude, Working intention, Retention, Rural areas, Health workforce, China

## Abstract

**Background:**

Attracting and recruiting health workers to work in rural areas is still a great challenge in China. The rural-oriented tuition-waived medical education (RTME) programme has been initiated and implemented in China since 2010. This study aimed to examine the attitudes of rural-oriented tuition-waived medical students (RTMSs) in Shaanxi towards working in rural areas and the related influencing factors.

**Methods:**

A cross-sectional survey was conducted in 2015 among 232 RTMSs in two medical universities from the first group of students enrolled in the RTME programme in Shaanxi. Descriptive and analytical statistics were used for the data analyses.

**Results:**

Of the 230 valid responses, 92.6% expressed their intentions of breaking the contract for working in rural township hospitals for 6 years after their graduation under the RTME programme. After the contract expired, only 1.3% intended to remain in the rural areas, 66.5% had no intention of remaining, and 32.2% were unsure. The factors related to a positive attitude among the RTMSs towards working in rural areas (no intention of breaking the contract) included being female, having a mother educated at the level of primary school or below, having a good understanding of the policy, having a good cognition of the value of rural medical work, and being satisfied with the policy. The factors related to a positive attitude of the RTMSs towards remaining in rural areas included being female, having a rural origin, having no regular family monthly income, having a father whose occupation was farmer, having a mother educated at the level of postsecondary or above, having the RTMSs be the final arbiter of the policy choice, having a good understanding of the policy, having a good cognition of the value of rural medical work, and being satisfied with the educational scheme.

**Conclusions:**

Related policy makers and health workforce managers may benefit from the findings of this study. Appropriate strategies should be implemented to stimulate the RTMSs’ intrinsic motivation and improve their willingness to work in rural areas and to better achieve the objectives of RTME policy. Meanwhile, measures to increase the retention of RTMSs should also be advanced.

**Electronic supplementary material:**

The online version of this article (10.1186/s12909-018-1209-z) contains supplementary material, which is available to authorized users.

## Background

The World Health Organization (WHO) defines health workers as all people engaged in actions with the primary intent of enhancing health [1]. The saying that there is no health without a workforce has been a universal truth. Health systems can function only with health workers; improving health service coverage and health outcomes depends on a fit-for-purpose and fit-to-practice health workforce [2]. With the continuously growing and ageing population and the continuously changing epidemiology and new technologies, the need for health workers will be expected to grow significantly in the coming decades. Meanwhile, a sufficient and qualified health workforce is absolutely essential to achieve the health-related targets of the sustainable development goals (SDGs) and universal health coverage [3].

However, the shortage and misdistribution of the health workforce have been global concerns affecting almost all countries, especially in rural and remote areas. In addition, the misdistribution between urban and rural areas has further led to more shortages of health workers in rural areas. Half of the world’s people currently live in rural and remote areas, but most health workers live and work in cities, which has further caused health inequity for the populations living in rural and remote locations [4]. Like many other countries, China is facing the severe challenges of an imbalance and inequity in its health workforce. In 2005, the doctor density in urban areas was more than twice that in rural areas (2.1 per 1000 people vs. 1.0 per 1000 people), and the nurse density showed a more than three-fold difference (1.7 per 1000 people vs. 0.5 per 1000 people) [5]. In 2011, these differences still existed and were basically the same as in 2005. The doctor density and nurse density were 3.00 and 3.29 per 1000 people in urban areas and 1.33 and 0.98 per 1000 people in rural areas, respectively. In addition, the health worker density was 7.90 per 1000 people in urban areas, which is more than twice that in rural areas (3.19 per 1000 people) in 2011. From 1985 to 2011, the inequalities of the health workforce distribution persisted, and most of them were due to inequalities in the rural-urban stratum [6]. Such health workforce inequalities matter greatly with respect to health outcomes and health inequity. For instance, a cross-county multiple regression analysis was conducted by using the 2000 census data in China and showed that the density of health workers was highly significant in explaining infant mortality [5]. Additionally, the inadequacy of health workers with a suitable skill set in rural hospitals has been a major impediment to the implementation and achievement of the policy goals of China’s healthcare reform [7–9].

The question of how to attract more health workers to work and live in rural and remote areas has drawn a great deal of attention from policy-makers, researchers, NGOs, etc. The WHO drew up a comprehensive set of strategies in 2010, which included interventions in education, regulation, financial incentives and professional and personal support, to help countries encourage health workers to live and work in remote and rural areas [4]. The new WHO Global Strategy on Human Resources for Health: Workforce 2030 (the Global Strategy) has also examined related evidence and provided some policy options and recommendations for transformative actions to effectively tackle the most significant health workforce challenges in the decades to come [10]. At the same time, various policy interventions have been implemented by national governments around the world, including both financial and non-financial incentives. Some interventions been found to effectively improve health workforce recruitment and retention in rural and remote areas, but the success of many of the interventions is still undetermined [11–19].

In response to these challenges of the health workforce in rural areas, China has put forward a series of health workforce development plans or policies. One of them was the rural-oriented tuition-waived medical education (RTME) programme initiated and promoted since 2010, which aimed to relieve the shortage of rural health workers in middle and west areas. In China, basically, there is a three-tier medical system in rural primary medical institutions that includes county-level hospitals, township hospitals and village clinics. As the main provider of rural primary healthcare services, township hospitals play an essential role in serving the majority (60%) of almost 850 million people in the rural areas of China [7, 9, 20, 21]. However, the township hospitals are severely understaffed [7]. These hospitals have difficulty recruiting sufficient health workers or can employ only health workers with very limited education [22]. Therefore, the RTME programme was implemented, and it aimed to enrol medical students mainly from rural areas to work in township hospitals for 6 years after they graduate. These medical students would sign a contract with the related departments in the government. The government would waive the related tuition for the students and gave them a certain amount of living allowance during their studies in designated medical universities [23]. The programme was carried out by the provincial governments specifically, and there were 23 provinces in middle and west areas in China that have begun to implement the programme since 2010. In 2010, the national programme recruited a total of 5000 rural-oriented tuition-waived medical students (RTMSs), and the number of RTMSs increased to 5810 in 2017. From 2010 to 2017, approximately a total of 43 thousand RTMSs enrolled in the national RTME programme in China.

Shaanxi, a western province with 46.08% of the 37.93 million population living in rural areas, put forward and implemented a 5-year undergraduate-level medical education programme under the national RTME programme since 2010. The first two medical universities qualified to enrol the RTMSs in Shaanxi were Xi’an Medical University and Yan’an University. The Shaanxi University of Chinese Medicine was added in 2011. According to the regulations of the RTME policy in Shaanxi, the three universities are eligible to enrol approximately 250 RTMSs in total every year.

Currently, a very limited number of studies have been conducted to evaluate the effect of the RTME programme. Therefore, this study aims to investigate these RTMSs enrolled in the programme and analyse their actual attitudes towards returning to work and remain in rural areas.

## Methods

### Study design

A cross-sectional survey was conducted during August and September 2015 via an email-based self-administered questionnaire developed jointly by the Xi’an Jiaotong University and the Xi’an Health and Family Planning Commission of Shaanxi Province. The employment guidance centres in the medical universities organized the survey and ensured the quality control.

### Study population

In July 2015, the first group of the RTMSs enrolled in 2010 under the 5-year undergraduate-level medical education programme in Shaanxi Province graduated from the first two medical universities and were ready to work. After 5 years of studies, they should have a more accurate understanding of the RTME programme and their intentions towards working in rural areas. The study was initially designed to investigate all of the 262 RTMSs in Xi’an Medical University and Yan’an University. However, only the RTMSs willing to participate in the study were chosen to fill in questionnaires.

### Questionnaire

A structured questionnaire was developed and used for the investigation. The questionnaire was anonymous and measured the participants’ attitudes subjectively. An additional file shows the questionnaire in more detail (see Additional file 1). The questionnaire consisted of four sections: (1) respondents’ general demographic characteristics, (2) policy cognition and policy choice motivation, (3) policy satisfaction, and (4) intention to break the contract and intention to remain after the contract expires. The demographic questions in section 1 asked the RTMSs to specify their gender, age, hometown or places they were brought up (rural versus urban), family monthly income (no regular income versus 3000 Yuan or below versus more than 3000 Yuan), father’s or mother’s occupation (farmer versus worker versus individual businessman versus other), and father’s or mother’s education (postsecondary or above versus senior high school versus junior high school versus primary school or below).

In section 2, the RTMSs were asked five questions: (1) Did you understand the conditions of the policy before enrolling? (very well or relatively well versus generally versus did not understand); (2) Why did you choose to enrol in the programme? (economic reasons versus guaranteed employment versus personal ambition versus other); (3) Who helped you to make the final decision of policy choice? (final arbiter was myself versus parents versus teacher); (4) Did you think the policy was effective for relieving the shortage of rural health workers? (cognition of policy’s effectiveness, yes versus unsure versus no); and (5) What was your cognition of the value of working in rural areas as a health worker? (very valuable or relatively valuable versus generally valuable versus no value).

In section 3, the RTMSs were asked about their satisfaction with the policy, including nine items: satisfaction with the policy publicity, satisfaction with the enrolment procedure, satisfaction with the employment contract, satisfaction with the educational scheme, satisfaction with the teachers, satisfaction with the living allowance, satisfaction with the work post after graduation, satisfaction with the government’s ability to handle problems of arranging employment, and satisfaction with the government’s speed of handling problems with arranging employment. All the nine items were measured using a Likert scale with five levels, from very unsatisfied to very satisfied.

In section 4, the RTMSs were asked two questions: (1) Did you have the intention to break the contract of the programme? (yes versus no) and (2) Would you remain and continue to work in rural areas as a health worker after the contract expires? (yes versus no versus unsure).

### Data analysis

SPSS software version 23 (IBM, Armonk, NY, USA) was used for the data analyses. The analyses consisted of three parts. First, descriptive statistics were used to describe the demographic characteristics of the RTMSs, policy cognition and policy choice motivation, policy satisfaction, intention to break the contract and intention to remain after the contract expires. These data were described as ‘percentages’ and ‘means’.

Second, univariate analyses were performed by cross-tabulations, using Pearson’s chi-square tests, to determine an association between the independent variables and dependent variables. In this study, the independent variables included demographic characteristics, policy cognition and policy choice motivation, and policy satisfaction; the dependent variables were intention to break the contract and intention to remain after contract expires. It is important to note that all the nine items related to policy satisfaction were arranged from scores of 1 to 5, which were regarded as continuous variables, so the study used the correlation analysis to discuss their correlations with the dependent variables.

Third, multivariate analyses were conducted to further determine the relationship between the independent variables and dependent variables. The independent variables selected in the regression models were those which were statistically significant in the univariate analyses and correlation analyses. For the dependent variables, the intention to break the contract was set as a binary variable (0 = no, 1 = yes); with regard to another dependent variable, i.e., the intention to remain after contract expires, since few medical students intended to remain, we transformed it into a binary variable (0 = no, 1 = yes/unsure). Therefore, binary logistic regression analyses were conducted ultimately. In Model 1, the impacts of the demographic characteristics were assessed. The factors of policy cognition and policy choice motivation were added in Model 2, and the policy satisfaction factors were further added in Model 3. A *P*-value less than 0.1 was regarded as statistically significant.

## Results

### Descriptive statistics

Among the 262 RTMSs enrolled in the first group of the Shaanxi RTME programme, 232 RTMSs were willing to participate in the investigation and completed the questionnaires. After performing quality control on the collected questionnaires, 2 questionnaires were rejected and 230 remained.

As summarized in Table 1, among the 230 RTMSs, 92.6% of them expressed that they intended to break the contract, which means 92.6% of the RTMSs actually did not want to go to rural township hospitals and work as rural health workers. Meanwhile, after the contract expired, only 1.3% of them intended to remain in the rural areas, 66.5% had no intention of remaining and 32.2% were unsure.

**Table 1:** Demographic characteristics of RTMSs (*N* = 230)

Variable	N (%)	Intention of breaking contract		*P*-value	Intention of remaining after contract expires			*P*-value
		Yes	No		Yes	No	Unsure	
Gender								
Male	77 (33.5)	75 (97.4)	2 (2.6)	0.038	0	61 (79.2)	16 (20.8)	0.011
Female	153 (66.5)	138 (90.2)	15 (9.8)		3 (2.0)	92 (60.1)	58 (37.9)	
Location of hometown								
Rural	189 (82.2)	174 (92.1)	15 (7.9)	0.386	2 (1.1)	120 (63.5)	67 (35.4)	0.064
Urban	41 (17.8)	39 (95.1)	2 (4.9)		1 (2.4)	33 (80.5)	7 (17.1)	
Family monthly income								
No regular income	135 (58.7)	126 (93.3)	9 (6.7)	0.453	0	84 (62.2)	51 (37.8)	0.001
≤ 3000 Yuan	53 (23.0)	50 (94.3)	3 (5.7)		0	40 (75.5)	13 (24.5)	
> 3001 Yuan	42 (18.3)	37 (88.1)	5 (11.9)		3 (7.1)	29 (69.0)	10 (23.8)	
Father’s occupation								
Farmer	184 (80.0)	170 (92.4)	14 (7.6)	0.589	3 (1.6)	116 (63.0)	65 (35.3)	0.019
Worker	32 (13.9)	31 (96.9)	1 (3.1)		0	30 (93.8)	2 (6.3)	
Individual businessman	6 (2.6)	5 (83.3)	1 (16.7)		0	4 (66.7)	2 (33.3)	
Other	8 (3.5)	7 (87.5)	1 (12.5)		0	3 (37.5)	5 (62.5)	
Father’s education								
Postsecondary or above	31 (13.5)	28 (90.3)	3 (9.7)	0.409	3 (9.7)	25 (80.6)	3 (9.7)	0.000
Senior high school	30 (13.0)	26 (86.7)	4 (13.3)		0	12 (40.0)	18 (60.0)	
Junior high school	121 (52.6)	115 (95.0)	6 (5.0)		0	84 (69.4)	37 (30.6)	
Primary school or below	48 (20.9)	44 (91.7)	4 (8.3)		0	32 (66.7)	16 (33.3)	
Mother’s occupation								
Farmer	163 (70.9)	149 (91.4)	14 (8.6)	0.571	3 (1.8)	105 (64.4)	55 (33.7)	0.011
Worker	20 (8.7)	20 (100)	0		0	18 (90.0)	2 (10.0)	
Individual businesswoman	16 (7.0)	15 (93.8)	1 (6.3)		0	15 (93.8)	1 (6.3)	
Other	31 (13.5)	29 (93.5)	2 (6.5)		0	15 (48.4)	16 (51.6)	
Mother’s education								
Postsecondary or above	12 (5.2)	12 (100)	0	0.001	1 (8.3)	8 (66.7)	3 (25.0)	0.052
Senior high school	15 (6.5)	10 (66.7)	5 (33.3)		0	6 (40.0)	9 (60.0)	
Junior high school	93 (40.4)	86 (92.5)	7 (7.5)		2 (2.2)	62 (66.7)	29 (31.2)	
Primary school or below	110 (47.8)	105 (95.5)	5 (4.5)		0	77 (70.0)	33 (30.0)	
Total	230	213 (92.6)	17 (7.4)		3 (1.3)	153 (66.5)	74 (32.2)	

#### Demographic characteristics

The mean age of the RTMSs was 25.14 years old. Table 1 presents the summary of RTMSs’ demographic characteristics. Among the 230 RTMSs, 66.5% were female, 33.5% were male. 82.2% were from rural areas, and 17.8% were from urban areas. More than half (58.7%) of the RTMSs were from families with no regular monthly income, 23.0% received 3000 Yuan or less per month, and only 18.3% received more than 3000 Yuan per month. A majority of the RTMSs’ fathers (80.0%) and mothers (70.9%) were farmers. Most of the RTMSs’ fathers (52.6%) had completed education through junior high school, a majority of the RTMSs’ (47.8%) mothers had completed only a primary school education or below, and a few students’ parents had a postsecondary or higher education.

#### Policy cognition and policy choice motivation

Table 2 presents the summary of the RTMSs’ policy cognition and their choice motivations. Before enrolling, more than three-quarters (75.7%) of the RTMSs did not understand the conditions of policy, while only 1.7% of them understood the policy very well or relatively well. The majority (56.1%) of the RTMSs chose the policy for economic reasons, and 37.5% chose it because of the guaranteed employment after graduation; however, no one chose it because of personal ambition. With regard to the final arbiter of the policy choice, the parents accounted for more than 70.4%, and the RTMSs themselves accounted for only 27.8%. Half of the RTMSs were unsure of the policy’s effectiveness regarding improving the rural health workforce shortage, 42.6% thought that it had no effectiveness, and only 7.4% thought it was effective. In terms of the value of rural medical work, 45.7% held the view that it was very or relatively valuable, 39.6% thought it was generally valuable; however, 14.8% thought that it had no value.

**Table 2:** Policy cognition and policy choice motivation of RTMSs (*N* = 230)

Variable	N (%)	Intention of breaking contract		*P*-value	Intention of remaining after contract expires			*P*-value
		Yes	No		Yes	No	Unsure	
Understanding the conditions of policy before enrolling								
Very or relatively well	4 (1.7)	3 (75.0)	1 (25.0)	0.002	0	1 (25.0)	3 (75.0)	0.097
Generally understand	52 (22.6)	43 (82.7)	9 (17.3)		2 (3.8)	31 (59.6)	19 (36.5)	
Don’t understand	174 (75.7)	167 (96.0)	7 (4.0)		1 (0.6)	121 (69.5)	52 (29.9)	
Policy choice motivation								
Economic reasons	129 (56.1)	120 (93.0)	9 (7.0)	0.661	1 (0.8)	91 (70.5)	37 (28.7)	0.126
Guaranteed employment	86 (37.4)	80 (93.0)	6 (7.0)		2 (2.3)	56 (65.1)	28 (32.6)	
Personal ambition	0	0	0		0	0	0	
Other	15 (6.5)	13 (86.7)	2 (13.3)		0	6 (40.0)	9 (60.0)	
Final arbiter of policy choice								
Myself	64 (27.8)	61 (95.3)	3 (4.7)	0.503	0	32 (50.0)	32 (50.0)	0.008
Patents	162 (70.4)	148 (91.4)	14 (8.6)		3 (1.9)	118 (72.8)	41 (25.3)	
Teacher	4 (1.7)	4 (100)	0		0	3 (75.0)	1 (25.0)	
Cognition of policy’s effectiveness								
Yes	17 (7.4)	13 (76.5)	4 (23.5)	0.003	0	9 (52.9)	8 (47.1)	0.180
No	98 (42.6)	88 (89.8)	10 (10.2)		3 (3.1)	63 (64.3)	32 (32.7)	
Unsure	115 (50.0)	112 (97.4)	3 (2.6)		0	81 (70.4)	34 (29.6)	
Cognition of value of rural medical work								
Very or relatively	105 (45.7)	92 (87.6)	13 (12.4)	0.021	3 (2.9)	62 (59.0)	40 (38.1)	0.069
Generally valuable	91 (39.6)	87 (95.6)	4 (4.4)		0	69 (75.8)	22 (24.2)	
No value	34 (14.8)	34 (100.0)	0		0	22 (64.7)	12 (35.3)	
Total	230	213 (92.6)	17 (7.4)		3 (1.3)	153 (66.5)	74 (32.2)	

#### Policy satisfaction

The mean score of the RTMSs’ satisfaction with the policy is displayed in Table 3. All nine mean scores related to policy satisfaction were lower than 3 points. Specifically, the highest score was satisfaction with teachers, which was 2.97 points; however, the score for satisfaction with the living allowance was the lowest at only 1.28 points.

**Table 3:** Policy satisfaction of RTMSs (*N* = 230)

Variable	Mean (S.D.)	Intention of breaking contract		Intention of remaining after contract expires	
		Correlation Coefficient	*P*-value	Correlation Coefficient	*P*-value
Satisfaction with policy publicity	2.19 (0.833)	- 0.188	0.002	0.017	0.784
Satisfaction with enrolment procedure	2.80 (1.097)	- 0.123	0.042	- 0.087	0.150
Satisfaction with employment contract	2.29 (1.108)	- 0.206	0.001	- 0.064	0.291
Satisfaction with educational scheme	2.18 (1.200)	- 0.189	0.002	0.303	0.000
Satisfaction with teachers	2.97 (1.144)	- 0.133	0.029	0.268	0.000
Satisfaction with living allowance	1.28 (0.547)	- 0.130	0.046	0.129	0.047
Satisfaction with work post after graduation	1.73 (0.946)	- 0.057	0.365	0.121	0.053
Satisfaction with ability to handle problems	1.48 (0.786)	- 0.077	0.228	0.149	0.019
Satisfaction with speed of handling problems	1.62 (0.799)	- 0.020	0.751	0.071	0.261

### Inferential statistics

We observed the following by conducting the univariate analyses. Factors associated with the RTMSs’ intentions to break the contract included gender, mother’s education, understanding the conditions of the policy before enrolling, cognition of the policy’s effectiveness, cognition of the value of working in rural areas, satisfaction with the policy’s publicity, satisfaction with the enrolment procedure, satisfaction with the employment contract, satisfaction with the educational scheme, satisfaction with the teachers, and satisfaction with the living allowance; these are displayed in Tables 1, 2 and 3 respectively. Factors associated with the RTMSs’ intentions to remain included gender, location of hometown, family monthly income, father’s occupation, father’s education, mother’s occupation, mother’s education, understanding the conditions of the policy before enrolling, final arbiter of policy choice, cognition of the value of working in rural areas, satisfaction with the educational scheme, satisfaction with the teachers, satisfaction with the living allowance, satisfaction with the work post after graduation, and satisfaction with the government’s ability to handle problems; these are displayed in Tables 1, 2 and 3 respectively.

After the binary logistic regression analyses, the following findings were further observed. Factors significantly associated with the RTMSs’ intentions to break the contract included gender, mother’s education, understanding the conditions of policy before enrolling, cognition of policy’s effectiveness, cognition of the value of working in rural areas, satisfaction with the enrolment procedure, satisfaction with the employment contract, satisfaction with the educational scheme, satisfaction with the teachers, and satisfaction with the living allowance; these are displayed in Table 4. Model 1 showed that male students were more likely to intend to break the contract than female students (OR = 3.821, *p* = 0.088); students whose mothers’ education included senior high school were more likely to have no intention of breaking the contract than those students whose mothers’ education included only primary school or below (OR = 0.101, *p* = 0.002). Model 2 further showed that, compared with those who did not understand the policy before enrolling, students who generally understood the policy were more likely to have no intention of breaking the contract (OR = 0.230, *p* = 0.031); compared with those who were unsure of the policy’s effectiveness regarding improving the rural health workforce shortage, students who were sure of the policy’s effectiveness, no matter whether it was effective or ineffective, were more likely to have no intention of breaking the contract (OR = 0.058 & 0.201, *p* = 0.003 & 0.057); and compared with those students who considered the rural medical work to be very or relatively valuable, students who thought it was generally valuable were more likely to intend to break contract (OR = 5.708, *p* = 0.022). Model 3 further showed that, with an increase in the degree of satisfaction with the enrolment procedure, employment contract, educational scheme and living allowance, students were more likely to have no intention of breaking the contract (OR = 0.379 & 0.446 & 0.287 & 0.276, *p* = 0.029 & 0.057 & 0.029 & 0.034); however, with an increase in the degree of satisfaction with teachers, students were more likely to intend to break the contract (OR = 4.533, *p* = 0.043).

**Table 4:** Binary logistic regression analyses on RTMSs’ intentions to break the contract for working in rural areas (*N* = 230)

Explanatory variables	Binary logistic regression (Base outcome: 0 = No; 1 = Yes)								
	Model 1			Model 2			Model 3		
	*B* (Std. Error)	Odds Ratio	*P*-value	*B* (Std. Error)	Odds Ratio	*P*-value	*B* (Std. Error)	Odds Ratio	*P*-value
Intercept	2.692 (0.476)			3.420 (0.773)			9.916 (2.697)		
Gender (Male)	1.340 (0.785)	3.821	0.088	1.870 (0.923)	6.489	0.043	1.097 (0.988)	2.996	0.267
Mother’s education (Reference value: Primary school or below)									
Senior high school	- 2.288 (0.728)	0.101	0.002	- 1.638 (0.883)	0.194	0.064	- 2.982 (1.359)	0.051	0.028
Understanding the conditions of policy before enrolling (Reference value: Don’t understand)									
Generally understand				- 1.470 (0.683)	0.230	0.031	- 1.295 (0.899)	0.274	0.150
Cognition of policy’s effectiveness (reference value: unsure)									
Yes				- 2.839 (0.950)	0.058	0.003	- 3.502 (1.541)	0.030	0.023
No				- 1.603 (0.841)	0.201	0.057	- 2.230 (1.206)	0.108	0.064
Cognition of value of rural medical work (reference value: very or relatively valuable)									
Generally valuable				1.742 (0.762)	5.708	0.022	2.347 (1.051)	10.454	0.026
Satisfaction with enrolment procedure							- 0.969 (0.445)	0.379	0.029
Satisfaction with employment contract							- 0.808 (0.425)	0.446	0.057
Satisfaction with educational scheme							- 1.249 (0.573)	0.287	0.029
Satisfaction with teachers							1.511 (0.748)	4.533	0.043
Satisfaction with living allowance							- 1.287 (0.606)	0.276	0.034

Method = Enter

In addition, factors significantly associated with the RTMSs’ intentions to remain after the contract expired included gender, location of hometown, family monthly income, father’s occupation, mother’s education, understanding the conditions of the policy conditions before enrolling, the final arbiter of the policy choice, cognition of the value of rural medical work, and satisfaction with the educational scheme; these are displayed in Table 5. Model 1 showed that male students were less likely to intend to remain in rural areas after the contract expired than female students (OR = 0.439, *p* = 0.027); rural students were more likely to intend to remain or be unsure than the urban students, rather than having no intention to remain (OR = 3.873, *p* = 0.055). Students whose families’ monthly income was 3000 Yuan or below were more likely to have no intention of remaining than those who came from the families with no regular monthly income (OR = 0.422, *p* = 0.048). Students whose fathers were workers were more likely to have no intention of remaining than those whose fathers were farmers (OR = 0.073, *p* = 0.037); students whose mothers’ education included only junior high school or primary school or below were more likely to have no intention of remaining than those whose mothers received a postsecondary education or above (OR = 0.059 & 0.052, *p* = 0.032 & 0.024). Model 2 further showed that, compared with those who understood the conditions of the policy very well or relatively well before enrolling, students who did not understood the policy were more likely to have no intention of remaining (OR = 0.059, *p* = 0.034); students whose parents were the final arbiters of the policy choice were more likely to have no intention of remaining after the contract expired than those who themselves were the final arbiters of the policy choice (OR = 0.242, *p* = 0.001). Compared with those who considered rural medical work to be very valuable or relatively valuable, students who thought it was generally valuable were more likely to have no intention of remaining (OR = 0.325, *p* = 0.008). Model 3 further showed that with an increase in the degree of satisfaction with the educational scheme, students were more likely to intend to remain or be unsure instead of having no intention (OR = 1.559, *p* = 0.028).

**Table 5:** Binary logistic regression analyses on RTMSs’ intentions to remain after the contract expires (*N* = 230)

Explanatory variables	Binary logistic regression (Base outcome: 0 = No; 1 = Yes/Unsure)								
	Model 1			Model 2			Model 3		
	*B* (Std. Error)	Odds Ratio	*P*-value	*B* (Std. Error)	Odds Ratio	*P*-value	*B* (Std. Error)	Odds Ratio	*P*-value
Intercept	1.094 (1.167)			5.981 (2.141)			3.233 (2.340)		
Gender (Male)	- 0.824 (0.373)	0.439	0.027	- 1.201 (0.412)	0.301	0.004	- 1.210 (0.443)	0.298	0.006
location of hometown (Rural area)	1.354 (0.705)	3.873	0.055	1.545 (0.762)	4.687	0.043	1.285 (0.819)	3.615	0.117
Family monthly income (reference value: no regular income)									
≤ 3000 Yuan	- 0.835 (0.422)	0.434	0.048	- 0.937 (0.482)	0.392	0.052	- 0.794 (0.529)	0.452	0.133
Father’s occupation (reference value: farmer)									
Worker	- 2.615 (1.254)	0.073	0.037	- 3.259 (1.496)	0.038	0.029	- 2.858 (1.435)	0.057	0.046
Mother’s education (reference value: postsecondary or above)									
Junior high school	- 2.824 (1.314)	0.059	0.032	- 3.272 (1.519)	0.038	0.031	- 3.279 (1.644)	0.038	0.046
Primary school or below	- 2.959 (1.307)	0.052	0.024	- 3.281 (1.523)	0.038	0.031	- 3.069 (1.647)	0.046	0.062
Understanding the conditions of policy before enrolling (Reference value: very or relatively well)									
Don’t understand				- 2.828 (1.333)	0.059	0.034	- 1.740 (1.382)	0.176	0.208
Final arbiter of policy choice (reference value: myself)									
Parents				- 1.420 (0.418)	0.242	0.001	- 1.444 (0.435)	0.236	0.001
Cognition of value of rural medical work (reference value: very or relatively valuable)									
Generally valuable				- 1.123 (0.423)	0.325	0.008	- 1.069 (0.461)	0.343	0.020
Satisfaction with educational scheme							0.444 (0.202)	1.559	0.028

Method = Enter

## Discussion

This study was conducted to evaluate the preliminary implementation effects of the RTME policy in Shaanxi, which analysed the RTMSs’ attitudes towards working in rural areas and identified the related predictors influencing their attitudes.

The age distribution of the RTMSs was found to be a mean age of 25.14 years with a standard deviation of 0.975 years. Approximately two-thirds (66.5%) of the RTMSs were female. More than four-fifths (82.2%) of the RTMSs were from rural areas. In addition, the study reported that 213 out of 230 (92.6%) RTMSs actually had a negative attitude towards working in rural township hospitals because they intended to break the RTME contract. This was different than the results of a local study on medical students conducted in Guangxi in China in 2015 [24]. In that study, 1523 out of 4669 (33%) medical students had a positive attitude towards working in rural township health centres, and 2574 (55%) students had a neutral attitude. Meanwhile, our study also reported that only 3 RTMSs (1.3%) intended to remain after the contract that working for 6 years in a township hospital expired, 74 (32.2%) were unsure, and 153 (66.5%) had no intention to remain. This means that there may be a high rate of mobility of the RTMSs from township hospitals to high-level health facilities or other institutions after the contract expires.

### Gender

In this study, female RTMSs were more likely to have a positive attitude towards working in rural township hospitals. Compared with male RTMSs, female RTMSs were less likely to intend to break the contract and more likely to intend to remain in rural areas after the contract expired. This result is consistent with a recent study conducted in five Asian countries in 2016, which showed that female medical students (2459/4432, 55.5%) were more likely to have a positive attitude towards working in rural areas than male medical students (1864/3539, 52.7%) [25]. This may because the rural male students have a greater desire to find a job and work in cities and thereby change their rural origins. However, the study conducted in Guangxi in China found that female students were less positive about rural medical service [24]; another survey of medical students in Ghana in 2011 also found that the female gender was associated with a reduced willingness to work in rural areas [26].

### Place of origin

A great deal of evidence has suggested that health workers with a rural background are more willing to work in rural areas [27]. Similarly, medical students with rural origins are more willing to work in rural areas compared with those with an urban background [28]. Our study has found that the rural RTMSs were more likely to intend to remain in the rural areas after the contract expired than those urban RTMSs, which is consistent with other related studies. A recent study conducted in Nepal in 2015 revealed that a rural hometown of medical students was nearly two times more likely to be associated with the choice of working in rural areas [29]. In addition, another local study conducted in China in 2016 showed that students from rural areas would be more likely to choose to work in communities than those from urban areas [30]. However, a contradictory finding was revealed by the study conducted in Ghana, which found that rural origin did not influence medical students’ willingness to work in rural areas after controlling for the intrinsic and extrinsic motivations and other demographic characteristics [26].

### Family factors

Parents are important factors with regard to their children’s attitudes towards work. The study conducted in Ghana revealed that high family or parental professional and educational status were consistently associated with a lack of willingness of medical students to work in rural areas [26]. In our study, three family factors that included the mother’s education, the father’s occupation and family monthly income were significantly associated with the RTMSs’ attitudes towards working and remaining in rural areas. When the mothers completed a low level of education, or the fathers’ occupation was worker rather than farmer, or the family monthly income was regular rather than absent, the student would be more likely to have a negative attitude towards working or remaining in rural areas. With regard to the family economic factor, the students from the families with regular incomes might give more priority to monetary income and working in rural areas cannot fulfil their drive [29]. In addition, an interesting finding in our study is that when parents were the final arbiters for their children to enrol in the RTME programme rather than the students choosing the policy and making the final decision by themselves, it was significantly reduced the RTMSs’ intentions to remain in the rural areas after the contract expired.

### Policy cognition and satisfaction

Our study highlighted the impacts of the RTMSs’ policy cognition and satisfaction on their attitudes towards working and remaining in rural areas. Our study found that 215 out of 230 RTMSs (93.5%) who chose the RTME programme did so because of extrinsic motivations, which included economic reasons and guaranteed employment after graduation. No one joined the programme because of personal ambition. However, many theoretical studies have suggested that intrinsic motivation, i.e., the desire to do something for its own sake, can have a strong effect on student’s job choice [26, 27, 31, 32]. Our study further verified this point. The RTMSs who did not understand the policy of the RTME programme before enrolling, or who were unsure of the policy’s effectiveness in improving the rural health workforce shortage, or who did not think that rural medical work was very or relatively valuable were more likely to have a negative attitude towards working and remaining in rural areas. Meanwhile, policy satisfaction (i.e., satisfaction with the enrolment procedure, employment contract, educational scheme, and living allowance) was significantly associated with an increased willingness to work in rural township hospitals after graduation. In particular, improving the RTMSs’ satisfaction with the educational scheme in the medical university seems to be very conducive to the students’ intentions of remaining in rural areas after the contract expired. Related studies have found that improving the curricular design (i.e., matching curricula with rural health needs and implementing early exposure and experience to rural medical work) could increase medical students’ interest in rural medical practice [24, 33, 34].

### Implications

The study has several implications. Related policy-makers may benefit from them. First, the programme could attract and enrol more medical students with a rural background due to the significant association between the intention to work in rural areas and rural origin. Second, family support and other extrinsic motivations are important; however, the RTMSs’ policy cognition and intrinsic motivations are more essential. The policy publicity should be strengthened, and the programme should improve the students’ cognition of the policy’s effectiveness and the value of rural medical work, which would cultivate and strengthen the students’ desire to perform rural medical work. Meanwhile, before enrolling, when selecting qualified medical students, the interview can be used to assess the applicants’ suitability as a non-academic measure, which may include their values, cognition, and other personal characteristics [24, 35]. Third, measures to increase the retention of the RTMSs rather than simply recruiting them should also be given greater attention [36], for instance, by providing appropriate and adequate local infrastructure and mentorship and setting competitive remuneration in rural medical institutions [26, 37]. More research is needed to determine the factors that influence the RTMSs’ intentions to remain after they have actually worked in the rural medical institutions. Fourth, the programme should also improve the RTMSs’ policy satisfaction by improving the efficiency of the enrolment procedure and contract signing and increasing the living allowance. In particular, the educational scheme can be redesigned and improved based on the RTMSs’ needs and the policy’s objectives.

### Limitations

First, as a cross-sectional study, it can only show a static picture that cannot thoroughly describe the changes between the actual behaviour and stated work intentions of the RTMSs. Second, the study could not capture the other factors affecting the RTMSs’ actual preferences in work, which means we could not examine whether the factors identified in this study truly affect the RTMSs’ choice in their actual work decisions. Third, the results of this study were based on a structured questionnaire and were mostly subjective assessments. This may not reflect the real ability of external factors to retain these RTMSs in the rural areas after the contract expires. In addition to individual factors, work-related factors (i.e., actual work conditions) also should be identified in further research. Finally, the effect or efficiency of the RTME policy could not be evaluated accurately based on just one cross-sectional survey of the first group of RTMSs. Under the above conditions, first, a larger sample size and more RTMSs should be involved in the survey to increase the study’s generalizability. Second, a longitudinal study is strongly recommended. Related follow-up studies should be conducted to measure what is actually happening in 2–3 years rather than intents of the RTMSs. Third, a qualitative study of the RTMSs as a supplement to the quantitative survey can be designed and implemented to facilitate a better understanding and assessment of the RTME programme.

## Conclusions

The majority of RTMSs report a negative attitude towards returning to work and remain in rural areas, which may have major implications for policy makers in relation to the RTME programme. Appropriate strategies should be put forward and implemented to better achieve the RTME policy objectives. In addition to recruitment under the RTME programme, attention should be devoted to the retention of the RTMSs in rural areas after the contract expires and the establishment of a reasonable flow mechanism for them as well as the development of more HRH policy support in China.

## Additional file

Additional file 1: Questionnaire of Rural-oriented Tuition-waived Medical Student Survey on their attitudes towards working in rural areas after graduation. (DOCX 96 kb)

## Abbreviations

HRH: Human resources for health
OR: Odds ratio
RTME: Rural-oriented Tuition-waived Medical Education
RTMS: Rural-oriented Tuition-waived Medical Student
WHO: World Health Organization
