# Supplementary material for: Attitude towards working in rural areas: a cross-sectional survey of rural-oriented tuition-waived medical students in Shaanxi, China
Source: BMC Med Educ. 2018 May 2;18:91. doi: 10.1186/s12909-018-1209-z (PMC5932863; doi:10.1186/s12909-018-1209-z)
Supplement: Supplementary file 1 — Questionnaire of Rural-oriented Tuition-waived Medical Student Survey on their attitudes towards working in rural areas after graduation. (DOCX 96 kb) [file 12909_2018_1209_MOESM1_ESM.docx]

**Questionnaire of Rural-oriented Tuition-waived Medical Student Survey on their attitudes towards working in rural areas after graduation**

| **Instruction:** Please choose one selection only.  **Background information:**  1. Gender: A. Male B. Female  2. Age: _____ years old  3. Origin: A. Urban B. Rural  4. Family monthly income (Yuan): A. No regular income B. 3000 Yuan or below C. More than 3000 Yuan  5. Father’s occupation: A. Farmer B. Worker C. Individual businessman D. Other  6. Father’s education: A. Postsecondary or above B. Senior high school C. Junior high school D. Primary school or below  7. Mother’s occupation: A. Farmer B. Worker C. Individual businesswoman D. Other  8. Mother’s education: A. Postsecondary or above B. Senior high school C. Junior high school D. Primary school or below  **Policy cognition and policy choice motivation:**  1. How well do you understand the policy of Rural-oriented Tuition-waived Medical Education (RTME) programme before enrolled?  A. Very or relatively well B. Generally C. don’t understand  2. Why do you choose to enrol in the programme of RTME?  A. Economic reasons B. Guaranteed employment C. Personal ambition D. other  3. Who help you to make the final decision to enrol in the RTME programme?  A. Myself B. My parents C. My teacher  4. Do you think the policy is helpful for relieving the shortage of rural health workers?  A. Yes B. Unsure C. No  5. What’s your cognition of working in rural areas as a health worker?  A. Very or relatively valuable B. Generally valuable C. No value  **Policy satisfaction:**   \| Policy Score \| 1 \| 2 \| 3 \| 4 \| 5 \| \| --- \| --- \| --- \| --- \| --- \| --- \| \| Very unsatisfied \| Unsatisfied \| General \| Satisfied \| Very satisfied \| \| Policy publicity \|  \|  \|  \|  \|  \| \| Enrolment procedure \|  \|  \|  \|  \|  \| \| Employment contract \|  \|  \|  \|  \|  \| \| Educational scheme \|  \|  \|  \|  \|  \| \| Teachers \|  \|  \|  \|  \|  \| \| Living allowance \|  \|  \|  \|  \|  \| \| Work post after graduation \|  \|  \|  \|  \|  \| \| Government’s ability to handle problems on arranging employment \|  \|  \|  \|  \|  \| \| Government’s speed of handling problems on arranging employment \|  \|  \|  \|  \|  \|   **Working intention in rural areas:**  1. Have you ever had the intention to break the contract of the RTME programme? (according to the contract, you need to work in township hospitals in rural areas for 6 years)  A. Yes B. No  2. Will you remain and continue to work in rural areas as a health worker after the contract expires?  A. Yes B. No C. Unsure |
| --- | --- | --- | --- | --- | --- | --- | --- | --- | --- | --- | --- | --- | --- | --- | --- | --- | --- | --- | --- | --- | --- | --- | --- | --- | --- | --- | --- | --- | --- | --- | --- | --- | --- | --- | --- | --- | --- | --- | --- | --- | --- | --- | --- | --- | --- | --- | --- | --- | --- | --- | --- | --- | --- | --- | --- | --- | --- | --- | --- | --- | --- | --- | --- | --- | --- |
